# Nanobody-Based Biologics for Modulating Purinergic Signaling in Inflammation and Immunity

**DOI:** 10.3389/fphar.2018.00266

**Published:** 2018-03-27

**Authors:** Stephan Menzel, Nicole Schwarz, Friedrich Haag, Friedrich Koch-Nolte

**Affiliations:** Institute of Immunology, University Medical Center Hamburg-Eppendorf, Hamburg, Germany

**Keywords:** nanobody, purinergic signaling, biologics, heavy chain antibody, antibody engineering

## Abstract

Adenosine triphosphate (ATP) and nicotinamide adenine dinucleotide (NAD^+^) are released as danger signals from cells during infection and sterile inflammation. In the extracellular compartment ATP is converted by CD39, CD73, and other ecto-enzymes into metabolites that modulate the activity of T cells and macrophages. While ATP mediates pro-inflammatory signals via P2X7 and other P2 receptors, adenosine triggers anti-inflammatory signaling via the adenosine 2a receptor (Adora2a) and other P1 receptors. The latter also plays a role in maintaining an immunosuppressive tumor microenvironment. NAD^+^ is converted by CD38, CD203 and other ecto-enzymes to the Ca^2+^ mobilizing messengers cyclic ADP-ribose and ADP-ribose, and to adenosine. Recent findings on the roles of CD38, CD39, CD73, CD203, P2X7, and Adora2a in inflammation and immunity underscore the potential of these proteins as drug targets. However, available small molecule inhibitors often lack specificity and mediate unwanted off-target toxicity. Nanobodies – single domain antibodies derived from heavy chain antibodies that naturally occur in camelids – display a propensity to bind functional epitopes not accessible to conventional antibodies. Like conventional antibodies, nanobodies and nanobody-based biologics are highly specific and have well-understood, tunable *in vivo* pharmacodynamics with little if any toxicity. Nanobodies thus represent attractive alternatives to small molecule inhibitors for modulating purinergic signaling in inflammation and immunity. Here we review recent progress made in developing nanobodies against key targets of purinergic signaling.

## Introduction

Purinergic signaling by extracellular ATP, NAD^+^ and their metabolites is recognized as an important regulatory mechanism in inflammation and immunity (Junger, 2011; Eltzschig et al., 2012; Idzko et al., 2014; Burnstock, 2016). The intact nucleotides and their common metabolite adenosine play pro- and anti-inflammatory roles, respectively. The ecto-enzymes and receptors that mediate purinergic signaling, therefore, represent promising targets for immunomodulatory drugs (Burnstock, 2017). A number of small molecule inhibitors are available that antagonize ionotropic (P2X) and metabotropic (P2Y) ATP-receptors, ATP- and NAD- hydrolyzing enzymes (CD38, CD39, CD73, CD203), and metabotropic (P1) adenosine receptors (Buque et al., 2016; Jacobson and Muller, 2016). Several of these show promising therapeutic benefit in animal models of inflammatory diseases and/or in animal tumor models (Buque et al., 2016; Burnstock, 2016). Recently, antibodies and nanobodies have emerged as a potent alternative class of therapeutics (Wesolowski et al., 2009; Krah et al., 2016; Steeland et al., 2016).

**FIGURE 1:**
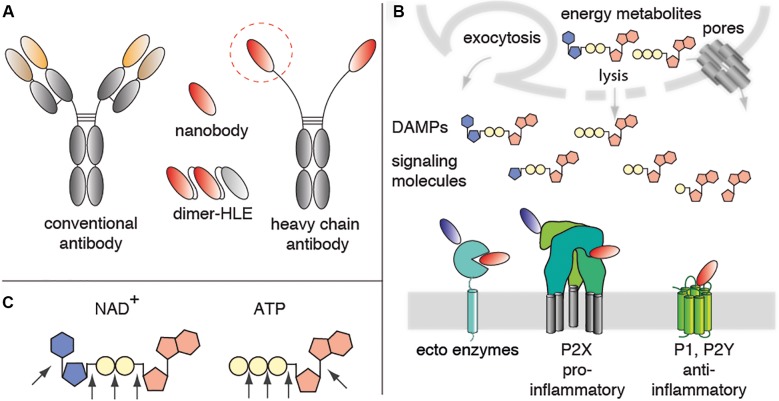
Mediators of purinergic signaling as potential nanobody targets. **(A)** Nanobodies correspond to the variable domain of heavy chain antibodies. These single domain antibodies often contain a long CDR3 that can extend into crevices on proteins that are not accessible to conventional antibodies. Nanobodies are highly soluble and are thus easily linked into bivalent and/or bispecific formats. For example, fusion of a homodimeric nanobody to an albumin-specific nanobody yields a half-life extended dimer (Dimer-HLE). **(B)** The energy metabolites ATP and NAD^+^ are released from cells during inflammation. In the extracellular space, these nucleotides function as danger associated molecular patterns (DAMPs) or signaling molecules. Immune cells express ecto-enzymes that convert pro-inflammatory ATP and NAD^+^ into anti-inflammatory adenosine. Immune cells also express ionotropic ligand-gated ion channels (P2X) and metabotropic GPCRs (P1, P2Y) that respond to ATP, NAD^+^ and their metabolites. These ecto-enzymes and purinergic receptors represent potential targets for immunomodulatory nanobodies. Antagonist nanobodies are illustrated schematically in red, potentiating nanobodies in blue. **(C)** Schematic diagram of NAD^+^, ATP and the linkages that are targets for ecto-enzymes.

Nanobodies are single domain antibody fragments derived from heavy chain antibodies naturally occurring in dromedaries, llamas, and other camelids (**Figure 1A**) (Hamers-Casterman et al., 1993; Muyldermans, 2013). In these animals, the IgG2 and IgG3 isotypes lack light chains and the CH1 domain. Nanobodies correspond to the variable domain of these heavy chain antibodies (also designated VHH). VHHs carry mutations that render them highly soluble in the absence of a paired light chain. VHHs often have longer CDR3 loops than the VH domain of conventional antibodies. These long CDR3 loops can mediate binding to hidden epitopes on target proteins such as the catalytic cavity of an enzyme, the ligand binding site of an ion channel or of a GPCR (Lauwereys et al., 1998; De Genst et al., 2006; Wesolowski et al., 2009). Because of their small size (15 kD, 3 nm), nanobodies generally show excellent tissue penetration. On the other hand, these small proteins pass the renal filtration barrier, accounting for a much shorter serum half life than that of conventional antibodies. Their robust, soluble single domain format, renders nanobodies amenable for genetic fusion to other protein domains. It is much easier to link two or more nanobodies into bi- or multivalent formats than the corresponding VH+VL domains of conventional antibodies. Linkage to an albumin-specific nanobody, for example, provides a simple strategy to extend the *in vivo* half life (Tijink et al., 2008). Nanobodies, thus represent promising tools to target the key players of purinergic signaling, in particular hidden functional epitopes of these proteins (**Figure 1B**).

## Nucleotide Metabolizing Ecto-Enzymes as Target for Nanobodies

Adenosine triphosphate (ATP) and nicotinamide adenine dinucleotide (NAD^+^) are released as danger signals from cells during infection and sterile inflammation (Haag et al., 2007; Junger, 2011; Eltzschig et al., 2012; Idzko et al., 2014). In the extracellular compartment ATP is converted by CD39, CD73, CD203 and related enzymes into ADP, AMP, and adenosine, metabolites that modulate the activity of T cells and macrophages. NAD^+^ is converted by CD38, CD73, and CD203 into nicotinamide and the Ca^2+^ mobilizing messengers cyclic ADP-ribose (cADPR) and ADP-ribose (ADPR), and finally to adenosine (**Figure 1C**). Extracellular NAD^+^ is also a substrate for the CD296 family of toxin-related ecto-enzymes that modify cell surface proteins by ADP-ribosylation.

### Nanobodies Targeting CD296/ARTC2.2

In response to NAD^+^ released from cells during inflammation, the ARTC2.2 ecto-enzyme ADP-ribosylates the P2X7 ion channel (Adriouch et al., 2008; Laing et al., 2010) and other cell surface proteins. ADP-ribosylation of P2X7 at R125 covalently attaches the weak ligand ADP-ribose close to the ATP binding pocket. Chronic activation of P2X7 leads to cell death. This phenomenon is designated NICD (Seman et al., 2003). Regulatory T cells, NKT cells, and tissue resident memory T cells (Trm) are particularly sensitive to NICD (Hubert et al., 2010; Rissiek et al., 2014; Fernandez-Ruiz et al., 2016).

ARTC2 ecto-enzymes are not expressed by human cells because the orthologous gene in humans and other primates is inactivated by premature stop codons (Haag et al., 1994). Notwithstanding, ARTC2.2 is the first immune cell ecto-enzyme for which potent, highly specific, antagonistic nanobodies have been developed (Koch-Nolte et al., 2007). Systemic application of ARTC2.2 -blocking nanobodies in mouse models have yielded important insights that may be relevant also to nanobodies targeting other mediators of purinergic signaling. Nanobodies reach and opsonize ARTC2.2 on the surface of immune cells much faster than conventional antibodies: After intravenous or intraperitoneal injection at moderate dosage (2 mg/kg), the nanobodies completely opsonized ARTC2.2 within 10 min after injection, whereas conventional antibodies achieved this only at 2 h after injection (Koch-Nolte et al., 2007; Hubert et al., 2010; Scheuplein et al., 2010). Conversely, unbound nanobodies were rapidly eliminated via renal infiltration while conventional antibodies and nanobody-based heavy chain antibodies were shown to have a much longer half life (Bannas et al., 2014, 2015). The effective functional blockade of ARTC2.2 enzyme activity by the injected nanobodies and nanobody-based heavy chain antibodies could readily be monitored on T cells recovered from lymphatic tissues of nanobody-injected mice: both formats effectively blocked ARTC2.2-catalyzed ADP-ribosylation of cell surface proteins and ARTC2.2-mediated NICD (Koch-Nolte et al., 2007; Hubert et al., 2010; Scheuplein et al., 2010). By introducing three amino acid substitutions into the Fc domain [LSF previously shown to mediate enhanced binding of IgG to the neonatal Fc-receptor (Ghetie et al., 1997)] blockade of ARTC2.2 *in vivo* could be extended to >1 week, even after injection of only a very low dose (0.2 mg/kg) of the nanobody-based heavy chain antibody LSF mutant (Scheuplein et al., 2010). In the NOD mouse model of genetically determined type 1 diabetes, weekly injections of this ARTC2.2-blocking heavy chain antibody resulted in a significant elevation of iNKT cell numbers in lymph nodes and spleen for at least 4 weeks after injection.

During the preparation of primary lymphocytes from spleen, lymph node, blood or liver, sufficient quantities of NAD^+^ are released from the mechanically stressed cells to permit ARTC2.2-catalyzed ADP-ribosylation of P2X7 and induction of NICD of Tregs, NKT, and Trm cells (Hubert et al., 2010; Rissiek et al., 2014; Fernandez-Ruiz et al., 2016). A single systemic injection of ARTC2.2-blocking nanobodies or nanobody-based heavy chain antibodies 30 min before sacrifice effectively prevents NICD of NKT cells and Tregs during cell preparation. Without such treatment ARTC2.2-expressing regulatory immune cells show poor survival during *in vitro* re-stimulation as well in adoptive transfer studies.

### Nanobodies Targeting CD38

CD38, the major NAD^+^-hydrolyzing ecto-enzyme, plays a decisive role in controlling the local levels of extracellular NAD^+^. CD38 is expressed by various hematopoietic cells, in particular by B cells and NK cells. CD38 hydrolyzes NAD^+^ to ADPR and cADPR, releasing nicotinamide. ADP-ribose is further hydrolyzed to phosphoriboside and AMP by members of the CD203 family of phosphodiesterases and eventually to adenosine by CD73. Two CD38-specific antibodies (daratumumab, isatuximab) have recently been licensed for the treatment of multiple myeloma, since these tumor cells often upregulate cell surface expression of CD38 to very high levels (van de Donk et al., 2017). The therapeutic benefit of these antibodies is thought to be mediated by their cytotoxic effects on tumor cells rather than by antagonizing the enzymatic activity of CD38. Recent evidence indicates that CD38 is also strongly upregulated by many solid tumors. It has been proposed that CD38 contributes to the immunosuppressive tumor microenvironment by hydrolyzing NAD^+^ (Horenstein et al., 2015). In line with this hypothesis, blocking CD38 enzyme activity in the tumor microenvironment may be a therapeutic strategy. Isatuximab was shown to inhibit the enzymatic activity of CD38, even though its binding site is far away from the NAD^+^ binding crevice, implying that this antibody acts by an allosteric mechanism (Deckert et al., 2014). Recently, 22 CD38-specific nanobodies were reported, two of which inhibited and two of which enhanced CD38 enzymatic activity (Li et al., 2016; Fumey et al., 2017). The results of epitope mapping and cocrystallization analyses indicate that these functional nanobodies, like isatuximab, act in an allosteric fashion rather than by direct binding to the active site. It is conceivable that the active site cleft of CD38 faces the plasma membrane and therefore is not easily accessible to antibodies or nanobodies. Some of the nanobodies have been shown to effectively target CD38 on human tumor cells in a mouse Xenograft model (Fumey et al., 2017) and some of these nanobodies bind independently of daratumumab and isatuximab and may, therefore, be useful for detecting cell surface CD38 in daratumumab- and isatuximab-treated patients (Oberle et al., 2017).

### Other Ecto-Enzymes as Potential Targets for Inhibitory Nanobodies

Similar to ARTC2.2 and CD38, other nucleotide metabolizing ecto-enzymes including CD39, CD73, CD203, and alkaline phosphatase represent promising targets for nanobody-based antagonists and/or agonists (Yegutkin, 2008; Deaglio and Robson, 2011; Horenstein et al., 2013; al-Rashida and Iqbal, 2014) Blocking the hydrolysis of nucleotides to adenosine is predicted to promote inflammatory responses and thus may represent viable therapeutic strategies in chronic infection and oncology. Conversely, promoting the conversion of extracellular nucleotides to adenosine may be beneficial in chronic inflammation. Many of these enzymes display deep active site crevices that are difficult to block with conventional antibodies but may be accessible for nanobodies. CD73 may present an exception from this theme. The crystal structures of CD73 in closed and open conformation revealed that this enzyme undergoes a dramatic conformational rearrangement upon AMP-binding (Knapp et al., 2012). This dramatic conformational shift may facilitate antibody mediated inhibition of CD73 by non-competitive mechanisms, e.g., by sterically blocking the shift in conformation, rather than occluding the active site (Geoghegan et al., 2016).

## Purinergic P1 and P2 Receptors as Targets for Nanobodies

ATP, NAD^+^ and their metabolites act as ligands for ionotropic (P2X) and metabotropic (P1, P2Y) receptors, many of which are expressed by immune cells (Burnstock and Boeynaems, 2014; Di Virgilio and Vuerich, 2015). Targeting these cell surface receptors with antagonistic and/or agonistic nanobodies thus might also provide novel therapeutic approaches in inflammation and immunity.

### Nanobodies Targeting P2X7

The P2X7 ion channel is expressed by monocytes and T cells and is gated upon binding of extracellular ATP, permitting influx of calcium (Ca^2+^) and sodium (Na^+^) and efflux of potassium (K^+^) (Bartlett et al., 2014; Di Virgilio et al., 2017). This triggers assembly of the inflammasome, release of the pro-inflammatory cytokine IL-1 β (IL-1β), ectodomain shedding of membrane proteins and externalization of phosphatidylserine (Ferrari et al., 2006; Giuliani et al., 2017). P2X7 is a potential therapeutic target in inflammatory diseases, such as glomerulonephritis, multiple sclerosis, and chronic pain (Bartlett et al., 2014; McInnes et al., 2014).

A set of 21 nanobodies was selected by phage display from llamas immunized with cDNA expression vectors encoding mouse and human P2X7 (Danquah et al., 2016). Six of eighteen nanobody families either blocked or enhanced activation of mouse P2X7 by both ATP and NAD^+^-mediated pathways and two of three nanobody families effectively blocked ATP-mediated gating of human P2X7. Dimerization via a flexible G4S linker enhanced the potencies of both, the antagonistic 13A7 nanobody and the potentiating 14D5 nanobody to low nanomolar/high picomolar IC_50_ values. Both nanobodies modulated P2X7 function when added either before or after the ligand ATP. Intriguingly, addition of the 13A7 blocked binding of the 14D5 and vice versa, indicating that these nanobodies either bind to overlapping epitopes or to alternative conformational states of P2X7. Genetic fusion of homodimeric nanobodies to the albumin-binding nanobody Alb8 markedly increased the *in vivo* half-life from hours to days. This trimeric nanobody-format was designated HLE-dimer (half-life extended αP2X7 dimer). A similar extension of the *in vivo* half life was achieved by fusing the nanobody monomers to the hinge and Fc domains of mouse or human IgG, thereby reconstituting a bivalent heavy chain antibody format. In two different mouse models systemic administration of the P2X7-antagonistic 13A7 HLE-dimer at a moderate dosage (1 mg/kg every 3 days) significantly ameliorated inflammation scores. In the model of DNTB-induced allergic contact dermatitis, 13A7 HLE-dimer reduced local inflammation as measured by ear weight and levels of the inflammatory cytokines IL-1ß and IL-6 to a similar extent as the corticosteroid Dexamethasone. Similarly, in the model of antibody-induced glomerulonephritis, the P2X7-antagonistic 13A7 HLE-dimer significantly reduced leukocyte infiltration of glomeruli and proteinuria. In a surrogate human inflammation model with endotoxin-treated blood samples, the human P2X7 antagonistic Dano1 nanobody effectively blocked ATP-induced release of IL-1β with subnanomolar IC_50_ values, i.e., at >1.000 fold higher potency than benchmark small molecule inhibitors of P2X7. Nanobody Dano1 thus is an excellent clinical candidate.

### Other Purinergic Receptors as Potential Targets for Inhibitory Nanobodies

Analogous to P2X7, P2X4, and P2X1 are expressed by immune cells and could be targets for nanobody-based antagonists and/or agonists (Burnstock, 2016). The P1 and P2Y receptors are GPCRs that respond to adenosine and purine nucleotides, respectively (Jacobson and Muller, 2016) and Adora2a and P2Y11 receptors are most relevant for inflammation and immunity (Sitkovsky et al., 2014; Young et al., 2016; Kennedy, 2017). Since these GPCRs have much smaller extracellular domains than the P2X receptors, inducing specific antibodies against GPCRs is much more challenging than in case of P2XRs. Notwithstanding, potent antagonistic nanobodies have been developed from llamas immunized with cDNA expression vectors encoding two non-purinergic lymphocyte GPCRs: CXCR4 and ChemR23, receptors for chemotactic proteins (Jahnichen et al., 2010; Claes et al., 2016; Peyrassol et al., 2016). It is thus not farfetched to propose that this strategy may also yield nanobodies targeting P1 and P2Y receptors.

## Advantages and Limitations of Nanobody-Based Biologics in Purinergic Pharmacology

Nanobodies directed against key players of purinergic signaling are valuable tools for research and diagnosis. Fluorochrome-conjugated nanobodies, for example, are well suited for flow cytometry, microscopy, and molecular imaging applications (Fumey et al., 2017). For example, nanobody JK36 recognizes a non-overlapping epitope on CD38 and can therefore detect cell surface CD38 by flow cytometry even in patients under treatment with daratumumab – where occupancy of CD38 by daratumumab prevents binding of most commercial diagnostic mAbs (Oberle et al., 2017). Owing to their small size, fluorochrome-conjugated nanobodies often provide much higher resolution images in fluorescence microscopy than conventional antibodies (Pleiner et al., 2015). Nanobodies have also proven useful as crystallization chaperones for 3D-structure analyses of GPCRs (Steyaert and Kobilka, 2011). On the other hand, native membrane protein-specific nanobodies usually do not work well in Western-Blot analyses, since their target epitopes are often destroyed by denaturing gel electrophoresis.

**Table 1** summarizes advantages and limitations of nanobodies vs. antibodies and small molecule drugs. In contrast to most small molecule drugs, antibodies and nanobodies generally are not orally available, i.e., they need to be administered by infusion or subcutaneous injection. High costs of clinical development and production still are handicaps of antibodies and nanobodies (Brekke and Sandlie, 2003). On the other hand, antibodies and nanobodies show exquisite specificity to the target molecule and – in contrast to small molecules – show little if any off-target side effects (Steeland et al., 2016). Nanobodies and antibodies are biodegradable and – in contrast to small molecules – do not suffer from conversion to toxic metabolites. While antibodies display uniform pharmacodynamics and a longer *in vivo* or serum half life than small molecules and nanobodies, their large size (150 kD) limits their penetration into solid tumors and other tissues. With a small size (15 kD), excellent tissue penetration and rapid renal filtration of unbound molecules, nanobodies, however, are exceptionally well suited for *in vivo* molecular imaging applications (Chakravarty et al., 2014; Bannas et al., 2015; Rashidian et al., 2015; Beghein and Gettemans, 2017).

**Table 1:** Advantages and limitations of nanobodies and antibodies vs. small molecule drugs in purinergic pharmacology.

	Antibodies	Nanobodies	Small molecules
Size	150 kD	15 kD	∼1 kD
Development costs	High	Moderate	Usually low
Administration	i.v., s.c.	i.v., s.c., aerosol, topical	oral, i.v.
Specificity	High	High	Variable
Off target adverse effects	None	None	P1, P2, kinases, ATPases, dehydrogenases
On target adverse effects	Depends on target	Depends on target	Depends on target
*In Vivo* half-life	Can be adjusted by Fc-engineering	Can be adjusted by PEGylation or fusion to albumin-specific Nb	Variable (usually short)
Metabolites	Non-toxic, biodegradable	Non-toxic, biodegradable	Potentially toxic
Tissue penetration	Slow	Excellent in periphery	Variable
Tissue specificity	Targetable (bi-specific Abs)	Targetable (bi- specific Nbs)	Variable
Albumin binding	Usually not	Via albumin-specific Nb to extend half life, usually no effect on potency	May reduce potency

Antibodies and nanobodies both show little if any capacity to cross the BBB. This can be an advantage for the therapeutic targeting of purinergic enzymes and receptors in inflammation and oncology outside the nervous system, because this limits on-target side effects in the central nervous system. The BBB, however, presents a limitation for therapeutic targeting inflammation and oncology in the CNS. If desired, passage of antibodies through the BBB can be enhanced by genetic fusion or conjugation to binding modules for the transferrin receptor and other transcytosis receptors (Farrington et al., 2014). Because of their small size and modular nature, nanobodies may be suited better for this strategy than conventional antibodies. The Fc-domain of conventional antibodies can mediate unwanted complement dependent and/or cell-mediated cytotoxicity (CDC, ADCC). Here, lack of an Fc domain can be an advantage for nanobodies. If the deletion of tumor cells or certain immune cell subsets is desired for therapeutic purposes, nanobodies can readily be fused to the Fc domain of conventional antibodies to generate a nanobody-based heavy chain antibody (75 kD). Such heavy chain antibodies are amenable for the full power of Fc-engineering technologies, i.e., substitution of amino acid residues that enhance/reduce *in vivo* half life and/or cytotoxicity (Liu et al., 2017).

Most purine metabolizing ecto-enzymes and purinergic receptors are not restricted to immune cells. Many of these enzymes and receptors are also expressed by cells of the cardiovascular system and the CNS. It will thus be important to monitor potential on-target side effects of the nanobodies that modulate their target on cells of other tissues. Owing to their high solubility and modular format, nanobodies can easily be linked to other nanobodies (Els Conrath et al., 2001; Bannas et al., 2017). This opens the opportunity to generate bi-specific biologics with improved cellular specificity, e.g., by linking a purinergic receptor-specific nanobody to a nanobody directed against a tissue differentiation antigen.

## Conclusion and Outlook

Nanobodies display a propensity to bind functional epitopes not accessible to conventional antibodies. Nanobodies that antagonize the purine-metabolizing ecto-enzymes ARTC2.2 and CD38 or the ATP-gated ion channel P2X7 have provided proof of concept for the notion that these small biologics represent attractive alternatives to small molecule inhibitors for modulating purinergic signaling in inflammation and immunity. It is thus not unlikely that caplacizumab, the first nanobody that is bound to reach the clinic next year, will pave the way for nanobody-based therapeutics also in the field of purinergic pharmacology.

## Author Contributions

FH and FK-N conceived the project. SM, NS, and FK-N wrote the manuscript. All authors reviewed and approved the manuscript.

## Conflict of Interest Statement

FH and FK-N receive a share of antibody sales via MediGate GmbH, a wholly owned subsidiary of the University Medical Center Hamburg-Eppendorf. SM and FK-N are co-inventors on patent applications on nanobody transgenic mice and/or CD38- or P2X7-specific nanobodies. The other author declares that the research was conducted in the absence of any commercial or financial relationships that could be construed as a potential conflict of interest.

## Abbreviations

ADCC: antibody-dependent cytotoxicity
ADP: adenosine diphosphate
ADPR: adenosine diphosphate ribose
AMP: adenosine monophosphate
ATP: adenosine triphosphate
BBB: blood brain barrier
CDC: complement-dependent cytotoxicity
GPCR: G-protein coupled receptor
HLE: half-life extended
Ig: immunoglobulin, IL-, interleukin
kD: kilodalton
NAD^+^: nicotinamide adenine dinucleotide
NICD: NAD^+^ induced cell death.
